# Which hospital workers do (not) want the jab? Behavioral correlates of COVID-19 vaccine willingness among employees of Swiss hospitals

**DOI:** 10.1371/journal.pone.0268775

**Published:** 2022-05-26

**Authors:** Ankush Asri, Viola Asri, Baiba Renerte, Franziska Föllmi-Heusi, Joerg D. Leuppi, Juergen Muser, Reto Nüesch, Dominik Schuler, Urs Fischbacher

**Affiliations:** 1 University of Konstanz, Konstanz, Germany; 2 Thurgau Institute of Economics, Kreuzlingen, Switzerland; 3 University of Zurich, Zurich, Switzerland; 4 Spital Schwyz, Schwyz, Switzerland; 5 Swiss Institute for International Economics and Applied Economic Research, University of St.Gallen, St.Gallen, Switzerland; 6 Kantonsspital Baselland, Liestal, Switzerland; 7 University of Basel, Basel, Switzerland; Centers for Disease Control and Prevention, UNITED STATES

## Abstract

In many countries, the current vaccination rates are stagnating, to the extent that vaccine hesitancy—the delay or refusal to take recommended vaccinations—forms a major obstacle to ending the COVID-19 pandemic. This tendency is particularly concerning when observed among healthcare workers who are opinion leaders on medical matters for their patients and peers. Our study surveys 965 employees of two large Swiss hospitals and profiles vaccine-hesitant hospital employees using not only socio-demographic characteristics, but also a comprehensive set of standard behavioral preference measures: (i) Big-5 personality traits, (ii) risk-, time- and social preferences, and (iii) perceived prevailing social norms. Using multinomial probit models and linear probability models, we find that vaccine-hesitant hospital employees are less patient and less likely to perceive vaccination as the prevailing social norm—in addition to replicating previously published socio-demographic results. Our findings are robust to a range of model specifications, as well as individual and situational covariates. Our study thus offers actionable policy implications for tailoring public-health communications to vaccine-hesitant hospital employees.

## Introduction

Widespread hesitancy about the SARS-CoV-2 coronavirus disease 2019 (COVID-19) vaccination is a major obstacle to ending the current pandemic. Vaccine hesitancy is especially concerning among hospital employees, firstly, because they themselves are often at increased risk of catching and transmitting the disease [1, 2]. Secondly, they have a potentially powerful influence on the vaccination decisions of their patients and, in a more informal context, their peers. Understanding the vaccination willingness of healthcare workers is thus crucial for promoting vaccine acceptance among the general public during the current COVID-19 pandemic and future infection disease crises. Indeed, the vaccination rates among healthcare workers have already been shown to correlate positively with recommending COVID-19 vaccination to their patients [3, 4]. Given this substantial influence of healthcare workers on the vaccine willingness of the wider population, it is important to understand the drivers behind their vaccine hesitancy and tailor public-health messaging accordingly.

Our study uses an exceptionally wide range of individual, situational, and behavioral measures to better understand vaccine-hesitant hospital employees, such as doctors, nurses and other employees who provide indirect care and services (e.g., technical support, sanitation specialists). We thus extend the previous efforts to understand COVID-19 vaccine hesitancy by linking vaccine willingness of frontline workers in two large Swiss hospitals to a considerably wider range of established personality, risk-preference, social-preference, time-preference and social-norm measures. Various public-health campaigns have already benefited from psychologically motivated approaches [5], and we hereby acknowledge the importance of understanding the complex social and behavioral factors influencing the public acceptance of science-based public-health recommendations [6–10]. There are good reasons to believe that different behavioral effects could play a role in the healthcare worker population in comparison to the general population, and Swiss hospital employees are an exemplary sample to study COVID-19 vaccine hesitancy. In particular, the vaccine approval process, rollout order and availability in Switzerland in combination with the particular timing of our survey allows to minimize the impact of some of the complex organizational and structural factors influencing vaccine uptake. According to the 5 As model, vaccination as a process can be divided into five steps: access, affordability, and awareness (“the supply side”) followed by acceptance and activation (“the demand side”) [11]. Our survey in two large Swiss hospitals was launched in the first half of January 2021—right after the Swiss health regulator approved the COVID-19 vaccine and administered the first vaccines in the second half of December 2020 and right before the healthcare workers themselves started receiving the vaccines on a routine basis (in Switzerland, healthcare workers were the second group to be vaccinated, following the elderly residents). This ensured that any uncertainty regarding the “supply side factors” of the COVID-19 vaccine could be minimized [12].

We rely on numerous previous studies to motivate our hypothesized drivers of vaccine willingness as summarized in Table 1. Although several studies with a general-population focus have examined both socio-demographic drivers and behavioral drivers of vaccine willingness, these effects within the healthcare worker population remain unclear. Within the general population, much of the existing literature on vaccine willingness focuses on the explicit reasons for choosing to comply or oppose vaccination programs [13–17] as well as the socio-demographic correlates of vaccine willingness, such as gender, education, income level and minority group [18–26]. Beyond that, vaccine willingness has been shown to be linked to various behavioral traits such as agreeableness (being sympathetic, warm), neuroticism (being anxious, easily upset) and conscientiousness (being dependable, self-disciplined) [13, 27, 28], altruistic preferences towards others [13, 29], trust preferences towards authority figures [30–33], as well as cognitive reflection [13, 34] and locus of control [13, 35], among others. The existing literature on vaccine hesitancy in general, beyond the COVID-19 vaccine, thus indicates that there are several behavioral profiles that distinguish the vaccine-acceptant from the vaccine-hesitant, which are likely to have similar effects on the COVID-19 vaccine acceptance.

**Table 1:** Summary of hypotheses.

Variable type	Variable (binary or standardized)	Effect on vaccine willingness
Individual	Female	Negative
	Younger	Negative
	Has higher education	Positive
	Healthcare worker	Positive
	Native Swiss	Mixed
Situational	Had COVID-19 infection	Positive
	Had COVID-19 contact outside work	Positive
	COVID-19 risk-group member	Positive
	Lives with a COVID-19 risk-group member	Positive
	Uses public transport	Mixed
	Traveled internationally	Positive
Personality	Extroverted	Mixed
	Agreeable	Mixed
	Conscientious	Positive
	Stable	Mixed
	Open	Mixed
Behavioral	Risk averse	Mixed
	Patient	Positive
	Future oriented	Positive
	Altruistic	Positive
	Reciprocal	Positive
	Trusting	Positive
	Perceives vaccination as social norm	Positive

The first published studies from the COVID-19 pandemic have already demonstrated that more vaccine hesitancy can be found in general population that is younger, more female, less educated, more minority, and with lower income [13, 36–42]. To classify the various impacts, several models suggest tailoring the intervention to the needs of the population, thus by implication, understanding the specific population in the greatest possible detail [43–45].

Within the healthcare worker population, COVID-19 vaccine hesitancy around the world—from China and Turkey to France and the USA—has been shown to be linked to various socio-demographic and opinion measures, such as gender (being female), education (having lower education and income levels) and age (mixed results), as well as vaccination history and attitudes, and perceived risk of infection [3, 46–59]. Several of these published studies, with a sample size of at least 500 healthcare workers each, also highlight concerns about vaccine safety and efficacy (e.g., side effects, speed of development, incentives of the pharmaceutical companies) and personal reasons (e.g., health conditions, religious beliefs) as the reasons for COVID-19 vaccine hesitancy among healthcare workers. Yet, it is still unclear which behavioral and personality traits are most strongly correlated with vaccine hesitancy when controlling for these known socio-demographic effects. It is thus important to explore this topic further because healthcare workers are influential sources of information for their patients and peers and can promote COVID-19 vaccination among these groups.

Note that throughout this paper, we refer to “behavioral” correlates when discussing validated measures of individual preferences commonly used in the behavioral economics literature. In other disciplines, these measures can also be referred to as psychological, attitudinal or perceptual determinants.

## Methods

Both socio-economic factors *and* personality traits and preferences can play a role for increasing acceptance and take-up of the readily accessible and affordable vaccines among the surveyed Swiss hospital employees. Our hypotheses are summarized in Table 1. In our study, we first elicit gender, age, education, profession and nationality representing individual characteristics and define the respective hypotheses. Second, we elicit infection, risk-group and travel related factors representing situational factors and define the respective hypotheses. Third, to cover further heterogeneity, we elicit the Big-5 personality traits—openness, conscientiousness, extroversion, agreeableness, and stability [60]—and approach these dimensions without defining directional ex-ante hypotheses, following the mixed results in the existing literature.

Finally, we measure several behavioral-preference dimensions and define the respective hypotheses(Table 1). First, we elicit the willingness to take risks using an incentivized measure [61] and acknowledge that risk preferences can play a mixed role in this context. Throughout this paper, we define “mixed” hypotheses whenever past evidence does not appear to suggest directionality in any direction. Second, we elicit time preferences as vaccination comes with short-term costs and future individual as well as societal benefits. We use two measures to capture respondent’s time preferences—patience and their future orientation—considering patience as a pre-condition for future-oriented behavior. We hypothesize that both, patience and future orientation, are positively correlated with vaccine willingness.

Third, given that vaccines protect both vaccinated individuals and the society, we argue that, depending on the context, vaccination as such could be interpreted as a prosocial act and elicit measures for three commonly used dimensions of social preferences—altruism, reciprocityand trust [62]. Fourth, we elicit beliefs about the vaccine decision of others in one’s region and hypothesize that the perceived share of people in one’s region wanting to get vaccinated is positively related to one’s own decision to get vaccinated similar to previous studies [63–66]. We hence interpret this as a perceived social norm which varies between individuals. This interpretation is motivated by the fact that Switzerland has never considered a mandatory vaccination mandate. All survey questions are taken from validated and well-established surveys; see a more detailed description below and S1 Table in S1 Appendix.

During the second COVID-19 wave in Europe, when vaccinations for healthcare professionals were just about to start in Switzerland, from mid-January until end-February 2021, we conducted an online survey of employees from two large hospitals in Switzerland. The hospitals are located in the Northwest of Switzerland near Basel and in the central region of Switzerland near Schwyz. The general aim of the larger survey was to examine the behavioral correlates of compliance with COVID-19 preventive measures including vaccination and mask-wearing, attitudes on COVID-19 policies and prevalence of COVID-19 infections (see Asri et al. [67] for behavioral correlates of mask-wearing).

The hospital employees were invited by mail and email to participate in the online survey on a voluntary basis. Informed consent was obtained from all participants. The online survey data collection was carried out in accordance with relevant guidelines and regulations prescribed by the Ethics Committee Northwest and Central Switzerland. The ethical approval for this study was waived by the same ethics committee because the project uses only anonymized survey data and the survey did not include any questions deemed sensitive by the committee. Overall, 965 respondents participated in the survey: 638 respondents from the relatively larger hospital near Basel (response rate 20%) and 327 respondents from the relatively smaller hospital near Schwyz (response rate 50%). The survey duration was approximately 15–20 minutes, and it was in German, given that the working language in both hospitals is German, and the hospital management confirmed that all hospital employees are sufficiently fluent in German. Survey participants had to be at least 18 years old and not belong to the groups of external doctors, staff providing on-demand service or pastoral care.

For our dependent variable, the hospital employees reported their willingness to get vaccinated in the online survey by answering the following question: “Assuming that the COVID-19 vaccination is available free of charge and approved in Switzerland, would you like to be vaccinated?” There werefour answer options: “Yes, as soon as it is available”, “Maybe later when I have enough information”, “No, never”, and “No response”. We thus consider our dependent variable as categorical and focus on the first three options excluding respondents who selected “No response”. Out of 965 respondents, 25 respondents (2.6%) had already been vaccinated with one shot of the COVID-19 vaccine. As getting vaccinated has been completely voluntary at the hospitals, we code these respondents as “Yes, as soon it is available”. 49 respondents (5%) selected “No response”. These respondents are not included in the analysis.

Corresponding to our research hypotheses, as independent variables of interest, we examine individual characteristics, situational factors, personality traits, preferences and the perceived willingness of others to get vaccinated (as a proxy for perceived social norm) as potential factors that could influence the hospital workers’ willingness to get vaccinated.

The examined individual characteristics include being female, being younger (below 45 years old), having higher education, working in healthcare (i.e., doctors and nurses), and being native Swiss. Situational factors include having been infected with COVID-19 according to self-report, having had contact with COVID-19 outside the hospital, belonging to a COVID-19 risk group, living with someone who belongs to a COVID-19 risk group, using public transport, and having traveled internationally outside of Switzerland within the last six months of the pandemic. These situational variables are included as they may influence the perceived risks of getting infected with COVID-19.

To examine the personality traits, we focus on the Big-5 extroversion, agreeableness, conscientiousness, stability and openness in our analysis. Following Gosling et al. [60], respondents are asked to indicate on a Likert scale from 1 to 7 to what extent the given 10 pairs of personality traits apply to them. Combining the responses then allows us to calculate to what extent a respondent is extroverted, agreeable, conscientious, stable, and open (see S1 Table in S1 Appendix).

We include a comprehensive set of behavioral preferences:Risk aversion, patience, future orientation, general trust, altruism and reciprocity. We use incentivized tasks for risk aversion (choosing between lotteries that vary in risk) following Gneezy and Potters [61] and for time preference (choosing a lower payment now or a higher payment later) following Andersen et al. [68]. For the preferences to be future oriented, trust, be altruistic and reciprocal, we use commonly used non-incentivized survey questions in which respondents self-report their preferences on a Likert scale from 0 to 10. All variables are standardized with respect to mean and standard deviation (see S1 Table in S1 Appendix). Lastly, we ask the survey respondents to estimate the percentage of people in their region who want to get vaccinated to extract a proxy measure of the perceived social norm. Thesummary statistics of all variables used in the analysis are provided in S1 Table in S1 Appendix. As pre-specified, we did not aim at representativeness of our sample and therefore do not use survey weights in the analysis.

The empirical analysis is divided into three parts: First, descriptive statistics comparing characteristics of the three groups; second, multinomial probit regression models to account for the simultaneous influence of various variables; and third, an analysis of the share of variance being explained by the different types of variables.

First, we describe the three groups according to their reported willingness to get vaccinated in terms of their individual characteristics, situational factors, personality traits, preferences and perceptions. We present these results in so-called radar graphs that always display the mean values for each of the three groups. We use two-sided t-tests for independent samples to compare the two most extreme groups, those who are not willing to get vaccinated with those who want to get vaccinated as soon as possible.

Second, we use a multinomial probit model to compare how the four different types of variables of interest correlate with the three categories of the dependent variable. Even though the answer options can be considered as ordered, we use a categorical interpretation because we expect different motives to be relevant for being hesitant (maybe later) and reluctant (never). We regress the willingness to get vaccinated in its three categories on the individual characteristics, situational factors, personality traits, preferences and perceived social norm. All regressions include hospital and survey-time fixed effects (i.e. week of the survey) to account for potential regional differences and developments over time with respect to the vaccination roll-out, in addition to our described variables of interest.

Given the categorical nature of the dependent variable, we use multinomial probit regressions for our main specifications [69]. For the ease of interpretation, we present marginal effects in the main text and in S4-S7 Tables in S1 Appendix. The interpretation of the marginal effect is as follows: A change in one unit of the independent variable increases/decreases the probability of selecting one of the three answer options by the marginal effect expressed in percentage points. For binary variables as listed under individual and situational factors, a one-unit-change corresponds to switching from 0 to 1. For behavioral traits and preferences, we used standardized variables with respect to mean and standard deviation ($z=\frac{x_{i}-\mu }{\sigma }$) and a one-unit change refers to an increase by one standard deviation. For instance, for each of the three answer options, it indicates that for an individual being female compared to non-female increases/decreases the probability to select one of the three answer options “No, never”/“Maybe”/“Yes” by X percentage points. Similarly, being one standard deviation more extroverted, increases/decreases the probability to select one of the three answer options “No, never”/“Maybe”/“Yes” by X percentage points. Note that the sum of the three marginal effects is always equal to 0.

Our robustness checks are shown in the S1 Appendix. S8-S11 Tables in S1 Appendix provide additional results using multinomial probit regressions with and without controls. S12-S15 Tables in S1 Appendix provide additional results using multinomial probit regressions with non-standardized preferences and non-standardized personality traits. The results remain qualitatively the same in all considered specifications.

Finally, we examine how much of the variance in choosing “No, never”, “Maybe later” and “Yes, as soon as available” is explained by the four dimensions—individual characteristics, situational factors, personality traits and preferences. We report the R-squared from linear probability regression models with three different dependent variables—each of them equal to 1 for each of the presented answer option and 0 otherwise. For instance, for “No, never”, the dependent variable is 1 if the respondent selected “No, never” and 0 if the respondent selected “Maybe later” or “Yes, as soon as available”. Correspondingly, the other two dependent variables for the answer options “Maybe later” and “Yes, as soon as available” are coded. The variables of interest include individual characteristics, situational factors, preferences, personality traits and perceived social norm. All regressions include hospital and time fixed effects.

## Results

Our sample of hospital employees consists of primarily female respondents (78%), native Swiss (70%), from different age groups ranging from 18 years to retirement age and with different levels of education. In our sample 51.3% of the respondents are nurses, 14.3% are administrative staff, 8.7% are doctors, 4.8% work as technical support staff and 20.3% as other support staff. At the time of the survey, 14.2% of the respondents report that they have had a COVID-19 infection and 20.4% report that they have had contact with an infected person outside work. We present the detailed summary statistics in S2 Table in S1 Appendix.

**Fig 1:**
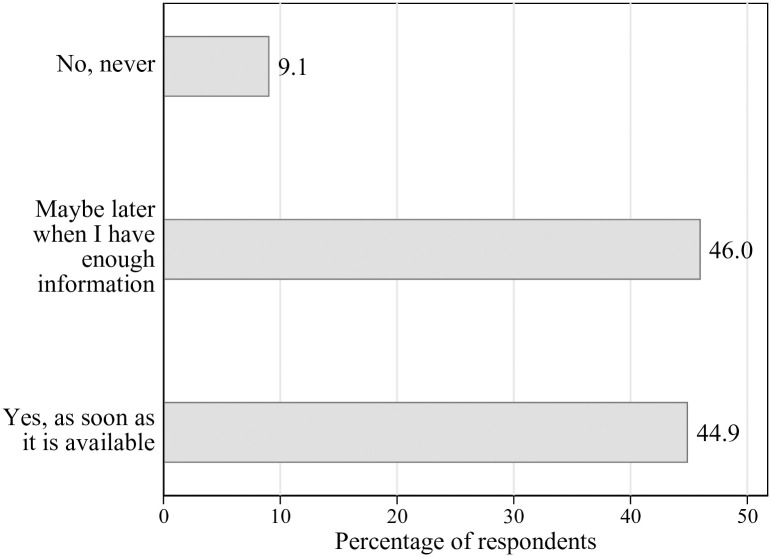
Willingness to get vaccinated. Survey question (n = 915): “Assuming that the COVID-19 vaccination is free of charge and approved in Switzerland, would you like to be vaccinated?”.

Starting with our dependent variable, the willingness to get vaccinated, Fig 1 shows that 9% of the respondents report that they never want to get vaccinated, 46% maybe want to get vaccinated later when they have enough information, and 45% indicate that they want to get vaccinated as soon as the vaccine is available.

### Descriptive analysis

**Fig 2:**
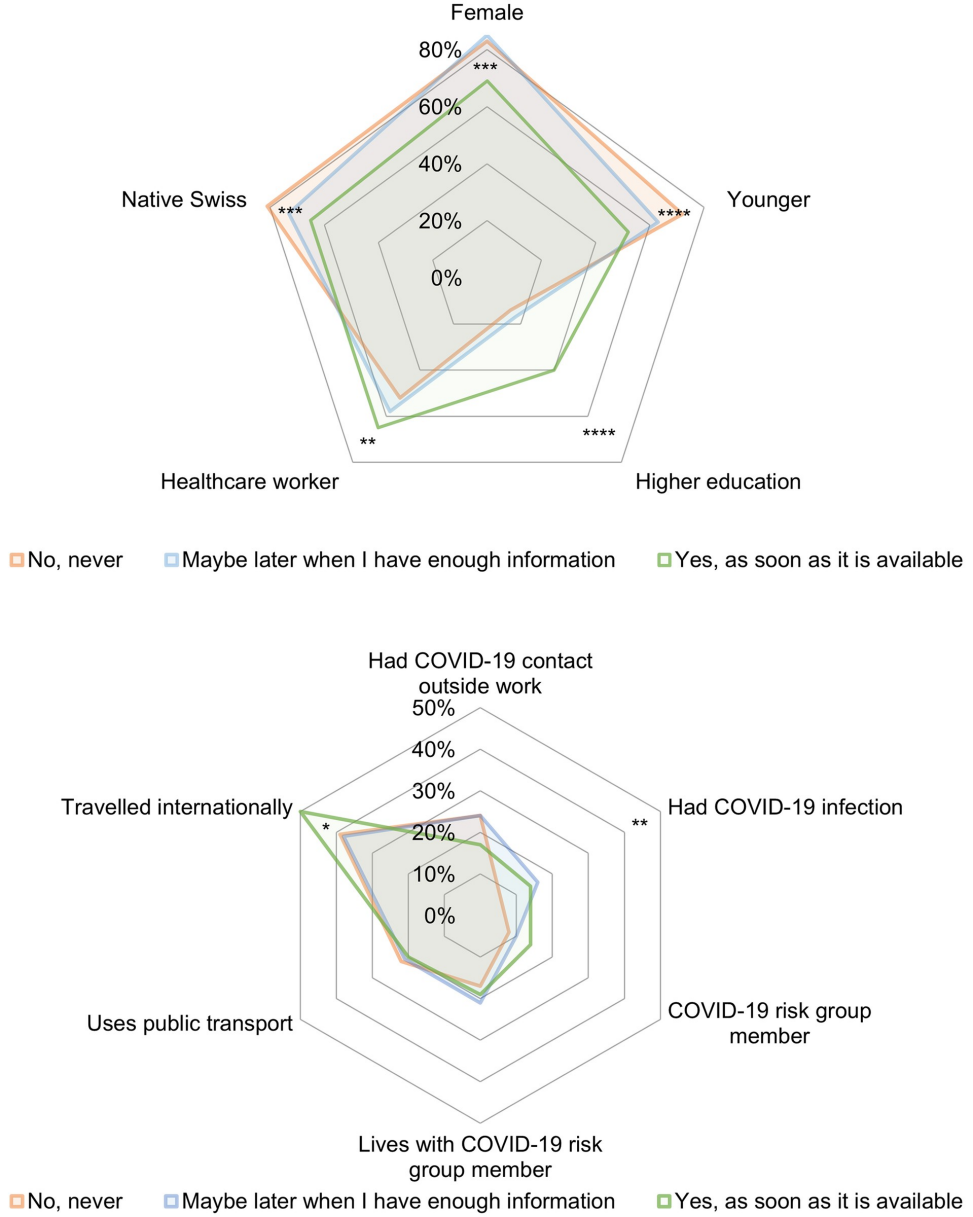
Descriptive statistics of individual and situational characteristics by vaccination willingness. (**a**) Individual characteristics (**b**) Situational factors. Survey question (n = 915): “Assuming that the COVID-19 vaccination is free of charge and approved in Switzerland, would you like to be vaccinated?” All variables are binary. For each binary characteristic, we show the percentage of respondents within each of the three groups. The statistical differences using two-sided t-tests for independent sub-samples between the “Yes, as soon as it is available” group and the “No, never” group are depicted as follows: * p < 0.1, ** p < 0.05, *** p < 0.01, **** p < 0.001.

**Fig 3:**
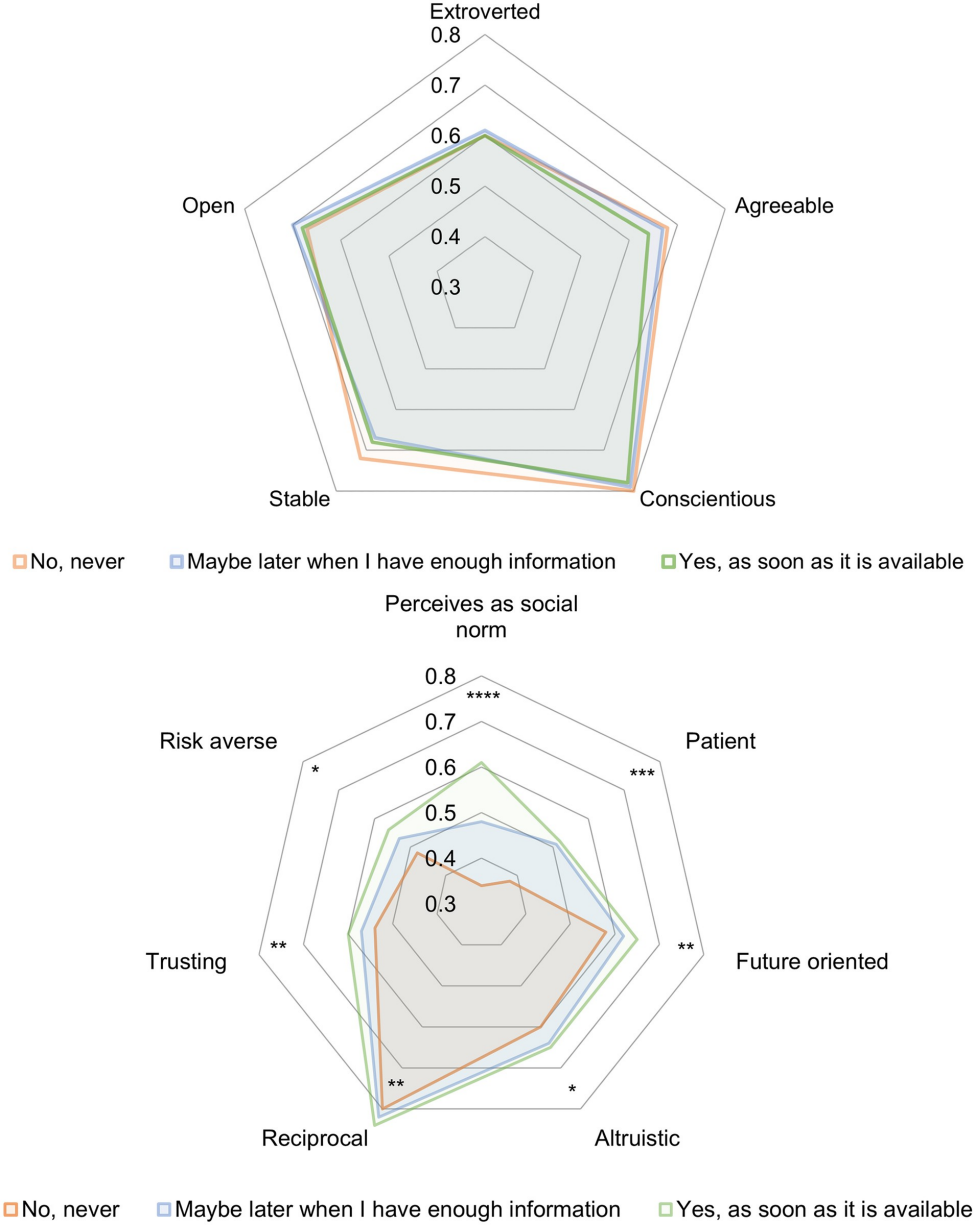
Descriptive statistics of personality traits, preferences and perceived social norm by vaccination willingness. (**a**) Personality traits (**b**) Preferences and perceived social norm. Survey question (n = 915): “Assuming that the COVID-19 vaccination is free of charge and approved in Switzerland, would you like to be vaccinated?” All variables are normalized and range from 0 to 1. For each characteristic, we show the mean value of the respondents within each of the three groups. The statistical differences using two-sided t-tests for independent sub-samples between the “Yes, as soon as it is available” group and the “No, never” group are depicted as follows: * p < 0.1, ** p < 0.05, *** p < 0.01, **** p < 0.001.

We use the survey data to examine how individuals with different willingness to get vaccinated differ in their individual characteristics, situational characteristics, personality traits and preferences as well as the perceived social norm. In Figs 2a, 2b, 3a and 3b, we use radar graphs to compare the profiles of the three groups descriptively in each of these four dimensions. Summary statistics of all variables are presented in S3 Table in S1 Appendix for each category of vaccine willingness in the S1 Appendix.

#### Individual characteristics

Corresponding to our hypotheses, as shown in Fig 2a, compared to the respondents who never want to get vaccinated, those who want to get vaccinated as soon as possible are on average less likely to be female (*p* < 0.01), less likely to be younger (defined as below 45 years old) (*p* < 0.001), more likely to have higher education (*p* < 0.001), more likely to be healthcare workers (*p* < 0.05). Compared to the respondents who never want to get vaccinated, those who want to get vaccinated as soon as possible are further less likely to be native Swiss (*p* < 0.01), for which we did not state a directional hypothesis.

#### Situational factors

In terms of situational factors, corresponding to our hypotheses, as shown in Fig 2b, compared to the respondents who never want to get vaccinated those who want to get vaccinated as soon as possible are more likely to have been previously infected with COVID-19 (*p* < 0.05) and more likely to have had traveled internationally within the last six months (*p* < 0.1). For other hypotheses on situational factors including having had COVID-19 contact outside work, being COVID-19 risk group member, living with COVID-19 risk group member and using public transport, we do not observe any significant differences.

We now turn to a comprehensive set of personality traits and preferences to examine the hospital workers’ willingness to get vaccinated through a behavioral lens.

#### Personality traits

The Big-5 personality traits, namely stability, agreeableness, conscientiousness, extroversion and openness on average do not differ between the respondents who never want to get vaccinated and those who want to get vaccinated as soon as possible. Fig 3a shows that descriptively there are no significant differences.

#### Preferences and the perceived social norms

We observe differences in terms of preferences as shown in Fig 3b. Corresponding to our hypotheses, compared to the respondents who never want to get vaccinated, those who want to get vaccinated as soon as possible are on average more patient (*p* < 0.01) and future-oriented (*p* < 0.05) than those who never want to get vaccinated. They further tend to perceive getting vaccinated as the social norm (*p* < 0.001) compared to those who never want to get vaccinated. The respondents who want to get vaccinated believe that 61% of the people in their region want to get vaccinated, the respondents who are unsure believe that 49% of the people in their region want to get vaccinated, and the respondents who do not want to get vaccinated believe that only 34% of the people in their region want to get vaccinated. While we did not have a clear hypothesis for risk, compared to the respondents who never want to get vaccinatedpreference, those who want to get vaccinated as soon as possible are on average slightly more risk averse (*p* < 0.1). In line with our hypotheses on social preferences, compared to the respondents who never want to get vaccinated, those who want to get vaccinated as soon as possible are slightly more altruistic (*p* < 0.1), more reciprocal (*p* < 0.05) and more trusting (*p* < 0.05).

### Regression analysis

**Fig 4:**
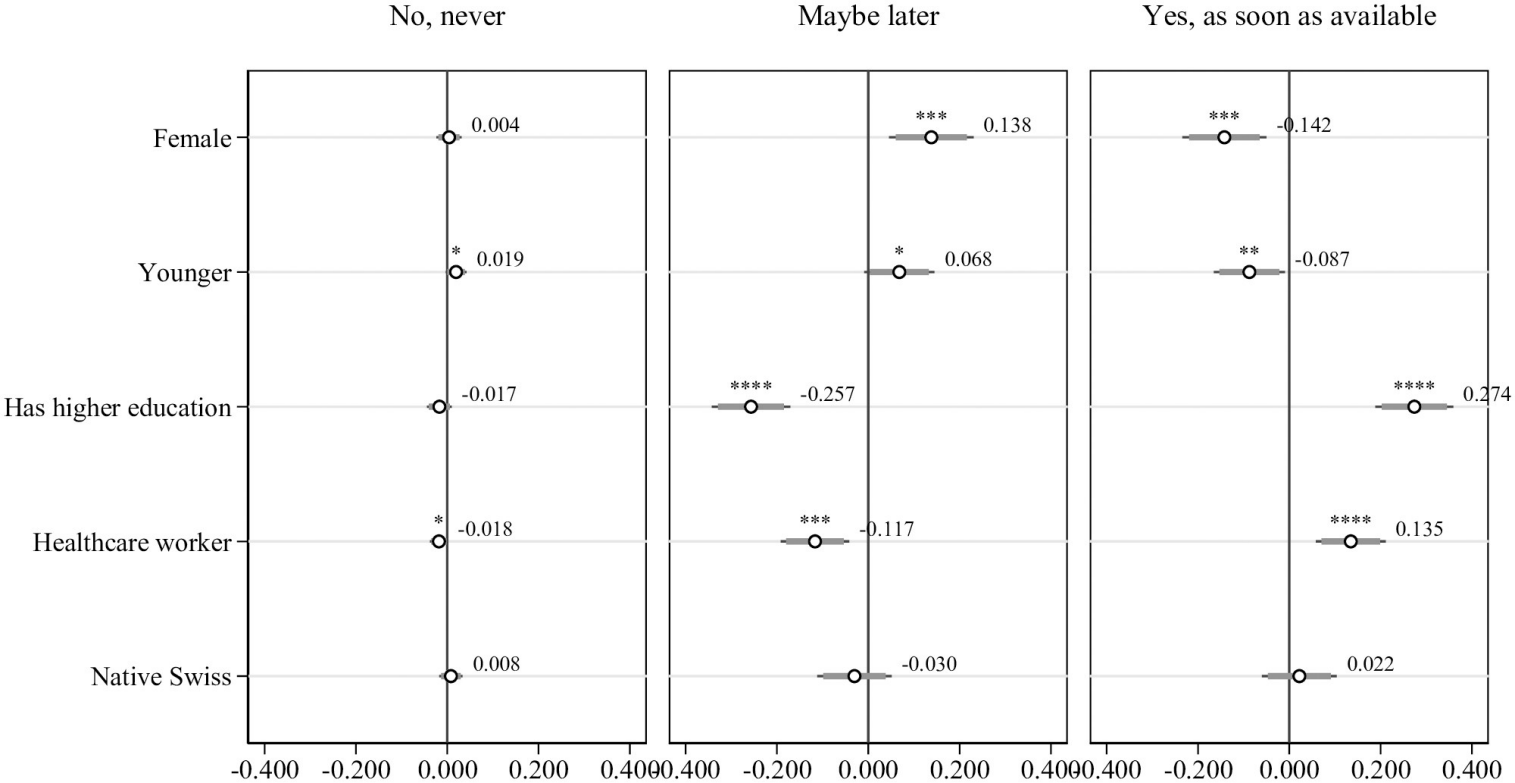
Multinomial probit regressions: Willingness to get vaccinated and individual characteristics, controlling for situational factors, Big-5 personality traits and preferences. Dependent variable is willingness to get vaccinated. Situational factors include belonging to COVID-19 risk group, living with someone belonging to COVID-19 risk group, having had contact with a COVID-19 infected person outside work, having been outside Switzerland, using public transport and having had a positive test result of COVID-19 in the past. Personality traits include extroversion, agreeableness, conscientiousness, stability and openness. Preferences include the level of trust in strangers, risk aversion, patience, future orientation, altruism and reciprocity and the perceived percentage of people in the region wanting to get vaccinated. All regressions include hospital and time fixed effects. Standard errors in parentheses, n = 915. * p < 0.1, ** p < 0.05, *** p < 0.01, **** p < 0.001.

**Fig 5:**
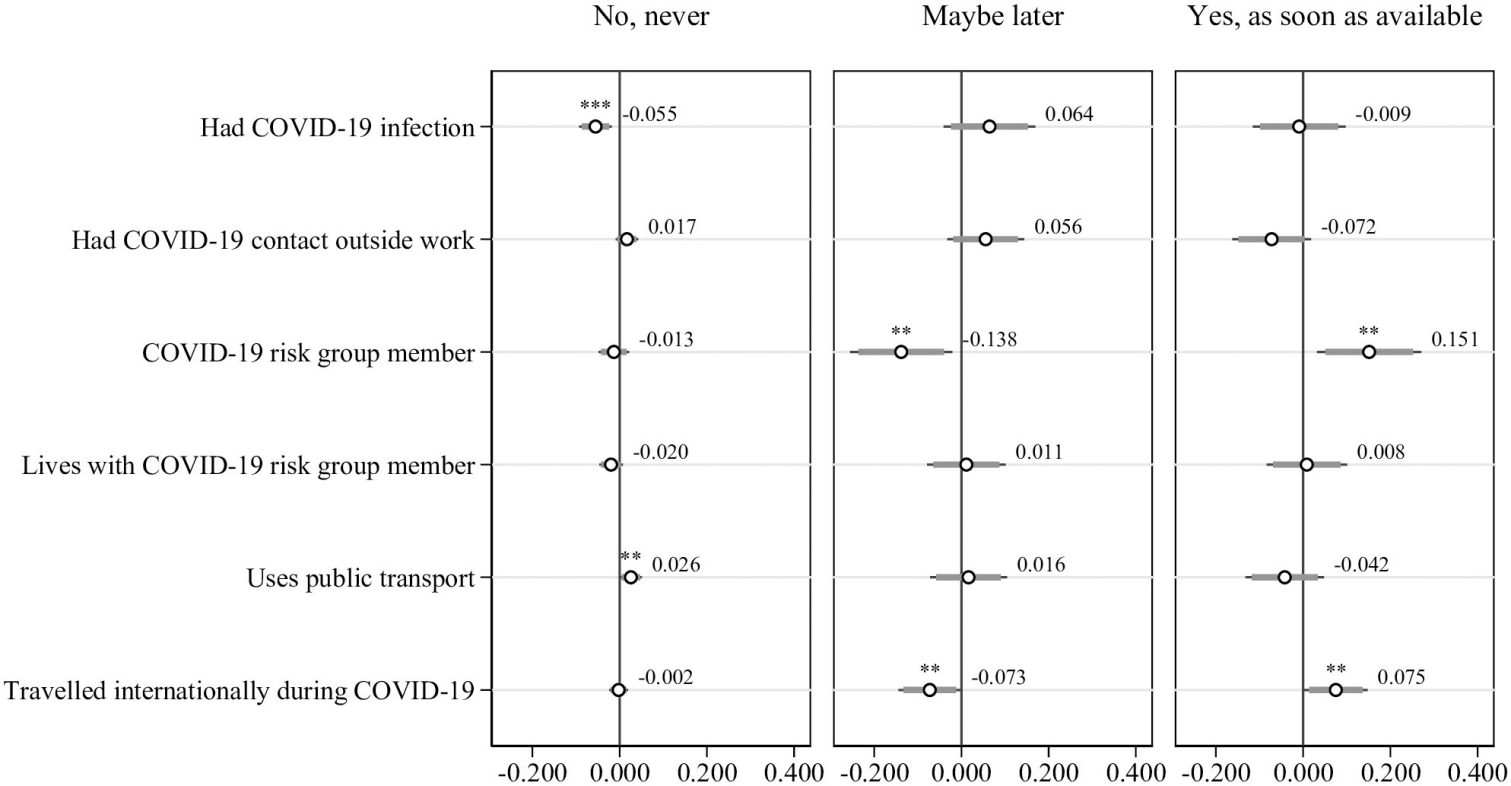
Multinomial probit regressions: Willingness to get vaccinated and situational factors, controlling for individual characteristics, Big-5 personality traits and preferences Dependent variable is willingness to get vaccinated. Individual characteristics include being female, being younger, education, being a healthcare worker, and being native. Preferences include the level of trust in strangers, risk aversion, patience, future orientation, altruism and reciprocity. Personality traits include extroversion, agreeableness, conscientiousness, stability and openness. All regressions include hospital and time fixed effects. Standard errors in parentheses, n = 915. * p < 0.1, ** p < 0.05, *** p < 0.01, **** p < 0.001.

**Fig 6:**
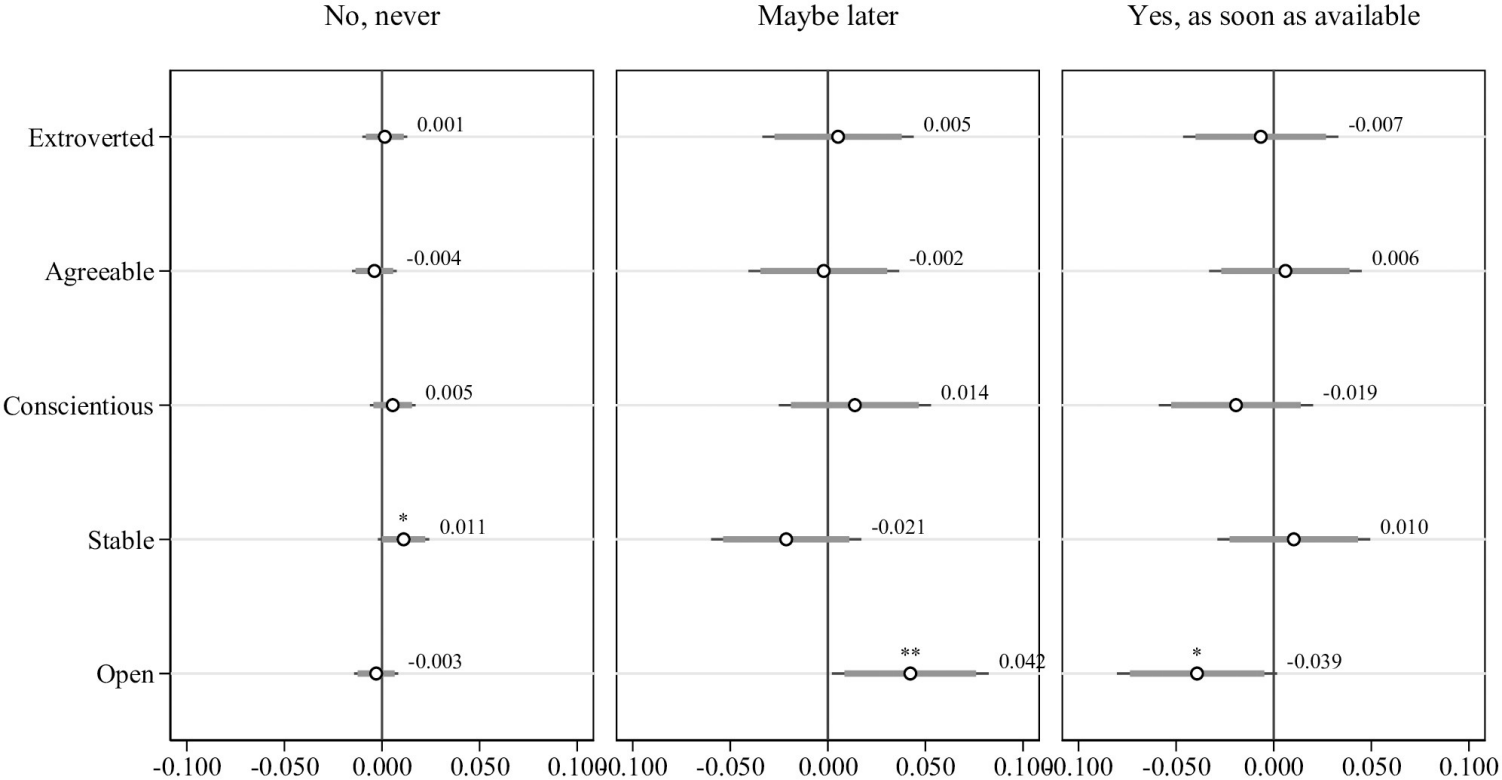
Multinomial probit regressions: Willingness to get vaccinated and Big-5 personality traits, controlling for individual and situational characteristics, and preferences. Dependent variable is willingness to get vaccinated. Individual characteristics include being female, being younger, education, being a healthcare worker, and being native. Situational factors include belonging to COVID-19 risk group, living with someone belonging to COVID-19 risk group, having had contact with a COVID-19 infected person outside work, having been outside Switzerland, using public transport and having had a positive test result of COVID-19 in the past. Preferences include the level of trust in strangers, risk aversion, patience, future orientation, altruism and reciprocity and the perceived percentage of people in the region wanting to get vaccinated. All regressions include hospital and time fixed effects. Standard errors in parentheses, n = 915. * p < 0.1, ** p < 0.05, *** p < 0.01, **** p < 0.001.

**Fig 7:**
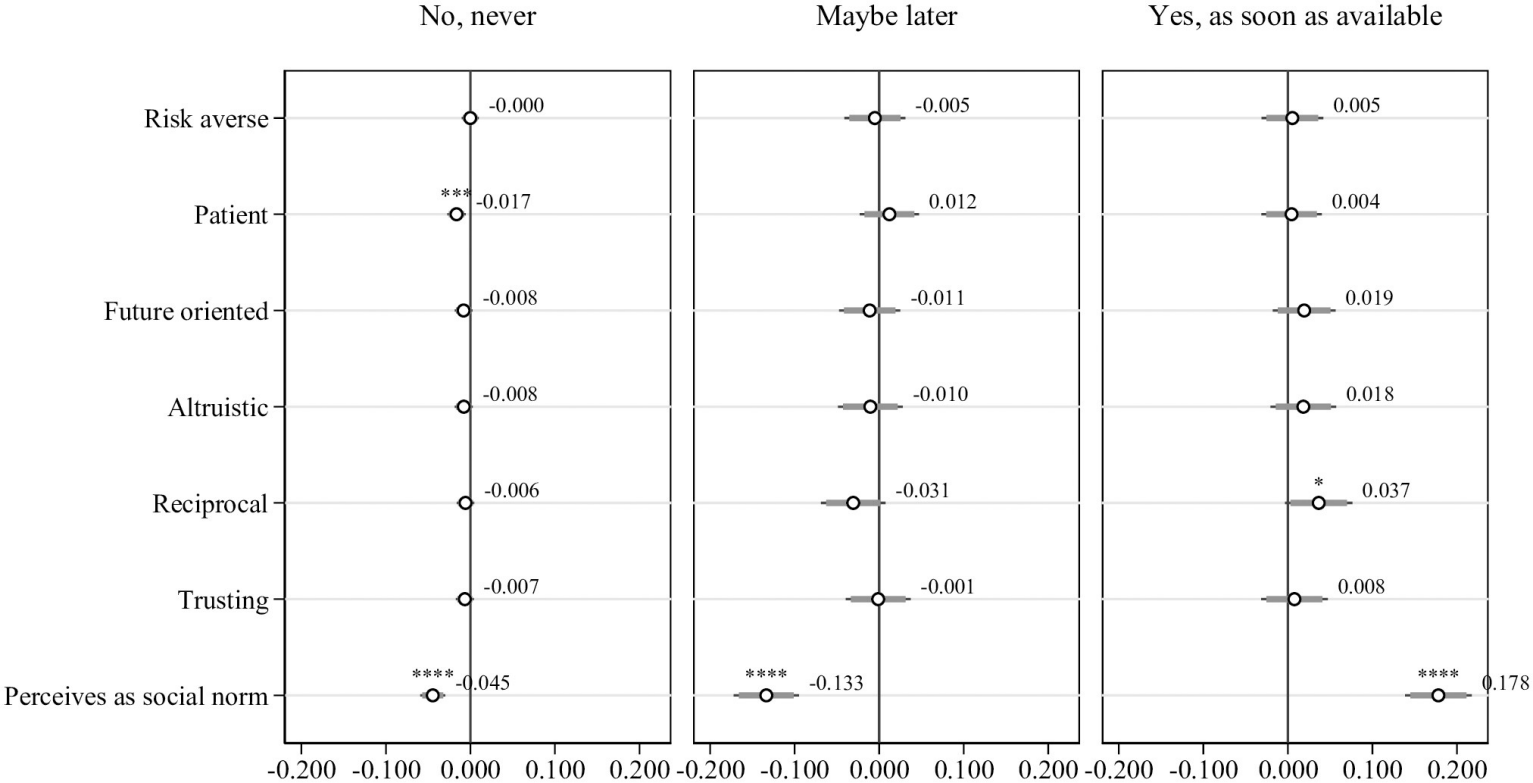
Multinomial probit regressions: Willingness to get vaccinated and preferences, controlling for individual and situational characteristics, and Big-5 personality traits. Dependent variable is willingness to get vaccinated. Individual characteristics include being female, being younger, education, being a healthcare worker, and being native. Situational factors include belonging to COVID-19 risk group, living with someone belonging to COVID-19 risk group, having had contact with a COVID-19 infected person outside work, having been outside Switzerland, using public transport and having had a positive test result of COVID-19 in the past. Personality traits include extroversion, agreeableness, conscientiousness, stability and openness. All regressions include hospital and time fixed effects. Standard errors in parentheses, n = 915. * p < 0.1, ** p < 0.05, *** p < 0.01, **** p < 0.001.

The descriptive comparisons presented above compare the respondents only with respect to mean values of one variable at a time and do not account for the possibility that some of the differences between the three groups may be related to other variables. In Figs 4 to 7, building on the four dimensions that could potentially influence hospital workers’ willingness to get vaccinated against COVID-19—individual characteristics, situational factors, personality traits, preferences and perceived social norm—we now proceed with multinomial probit regression analysis to examine which variables correlate with thehospital employee’s choice of“No, never”, “Maybe later” or “Yes, as soon as available” after controlling for other variables. Throughout the description of regression results, we present marginal effects. Therefore, the interpretation is always for one-unit change in the independent variable of interest increasing/decreasing the probability of choosing one answer option in percentage points (i.e., from 0 to 1 for binary variables and a one-standard-deviation change for continuous standardized variables).

#### Individual characteristics

Corresponding to our research hypotheses above, after controlling for other covariates, the willingness to get vaccinated is negatively correlated with being female and being younger but positively correlated with higher education and working as healthcare worker (Fig 4). Females compared to non-females are 13.8 percentage points more likely to answer “Maybe later” (*p* < 0.01) and 14.2 percentage points less likely to answer “Yes, as soon as available” (*p* < 0.01). Younger respondents compared to older respondents are 1.9 percentage points more likely to respond “No, never” (*p* < 0.1), 6.8 percentage points more likely to respond “Maybe later” (*p* < 0.1) and 8.7 percentage points less likely to respond “Yes, as soon as available” (*p* < 0.05). Highly educated hospital employees compared to not-highly-educated employees are 25.7 percentage points less likely to indicate “Maybe later” and 27.4 percentage points more likely to indicate “Yes, as soon as available” (for both *p* < 0.01). Healthcare workers (i.e., nurses and doctors) compared to other hospital employees (i.e., administrative, technical and other support) are 1.8 percentage points less likely to indicate “No, never” (*p* < 0.1), 11.7 percentage points less likely to indicate “Maybe later” and 13.5 percentage points more likely to indicate “Yes, as soon as available” (for both *p* < 0.01). There is no significant difference between native Swiss respondents and non-native respondents when we control for other covariates. In this specification, we control for situational factors, Big-5 personality traits, preferences and the perceived social norm. S4 Table in S1 Appendix shows the corresponding regression results.

#### Situational factors

Further, examining the situational factors that could influence the willingness to get vaccinated, we find that in line with our hypotheses, the willingness to get vaccinated is positively correlated with having been infected with COVID-19, belonging to a COVID-19 risk group and having traveled internationally within the last six months of the pandemic (Fig 5). It is further negatively correlated with using public transport for which we did not have a directional hypothesis. The respondents who have had a COVID-19 infection, compared to those who have had not, are 5.5 percentage points less likely to indicate “No, never” (*p* < 0.01), those who are a COVID-19 risk group member, compared to those who are not, are 13.8 percentage points less likely to indicate “Maybe later” (*p* < 0.05) and 15 percentage points more likely to indicate “Yes, as soon as available” (*p* < 0.05). The respondents who use public transport, compared to those who do not, are 2.6 percent more likely to indicate “No, never” and those who have traveled internationally since the beginning of the pandemic, compared to those who have not, are 7.3 percentage points less likely to indicate “Maybe later” and 7.5 percentage points more likely to indicate “Yes, as soon as available” (*p* < 0.05). Given that 9% of the respondents selected “No, never”, 46% selected “Maybe later” and 45% selected “Yes, as soon as available”, these correlations are also significant in size. S5 Table in S1 Appendix shows the corresponding regression results.

#### Personality traits

Personality traits seem to play a more limited—although still significant—role in predicting the willingness to get vaccinated as shown in Fig 6. We observe a suggestive correlation with the Big-5 measure of stability and a slightly stronger correlation with the Big-5 measure of openness. Being more stable is associated with a 1.1 percentage points increase in the likelihood of refusing the vaccine (*p* < 0.1) and being more open is associated with a 4.2 percentage points higher likelihood to select “Maybe later” (*p* < 0.05) and a 3.9 percentage points lower likelihood to indicate “Yes, as soon as available” (*p* < 0.1). Other personality traits do not seem to predict the vaccination willingness. S6 Table in S1 Appendix shows the corresponding regression results.

#### Preferences and the perceived social norms

Finally, in terms of behavioral preferences, corresponding to our hypotheses, the regression results also show that respondents who are morepatient are more willing to get vaccinated. Further, as hypothesized, more reciprocal respondents are more likely to express that they are willing to get vaccinated (Fig 7). Being more patient is associated with a 1.7 percentage points lower likelihood of choosing “No, never”(*p* < 0.01) and being more reciprocal is associated with a 3.7 percentage points higher probability of choosing “Yes, as soon as available” (*p* < 0.1). Regarding the perceived social norm, hypothesized to be positively correlated with the willingness to get vaccinated, our regression results show a very strong positive correlation—even after controlling for all the above-mentioned individual characteristics, situational factors, personality traits and preferences. Perceiving a higher share of the people in their region wanting to get vaccinated is associated with a 17.8 percentage points higher likelihood of selecting “Yes, as soon as available”, a 13.3 percentage points lower likelihood of selecting “Maybe later”, and a 4.5 percentage points lower likelihood of selecting “No, never” (*p* < 0.001 for all estimates). Note thatthe here presented regression results for behavioral preferences remain similar if we exclude the social norm variable from the regressions. S7 Table in S1 Appendix shows the corresponding regression results.

### Analysis of explained variance

**Fig 8:**
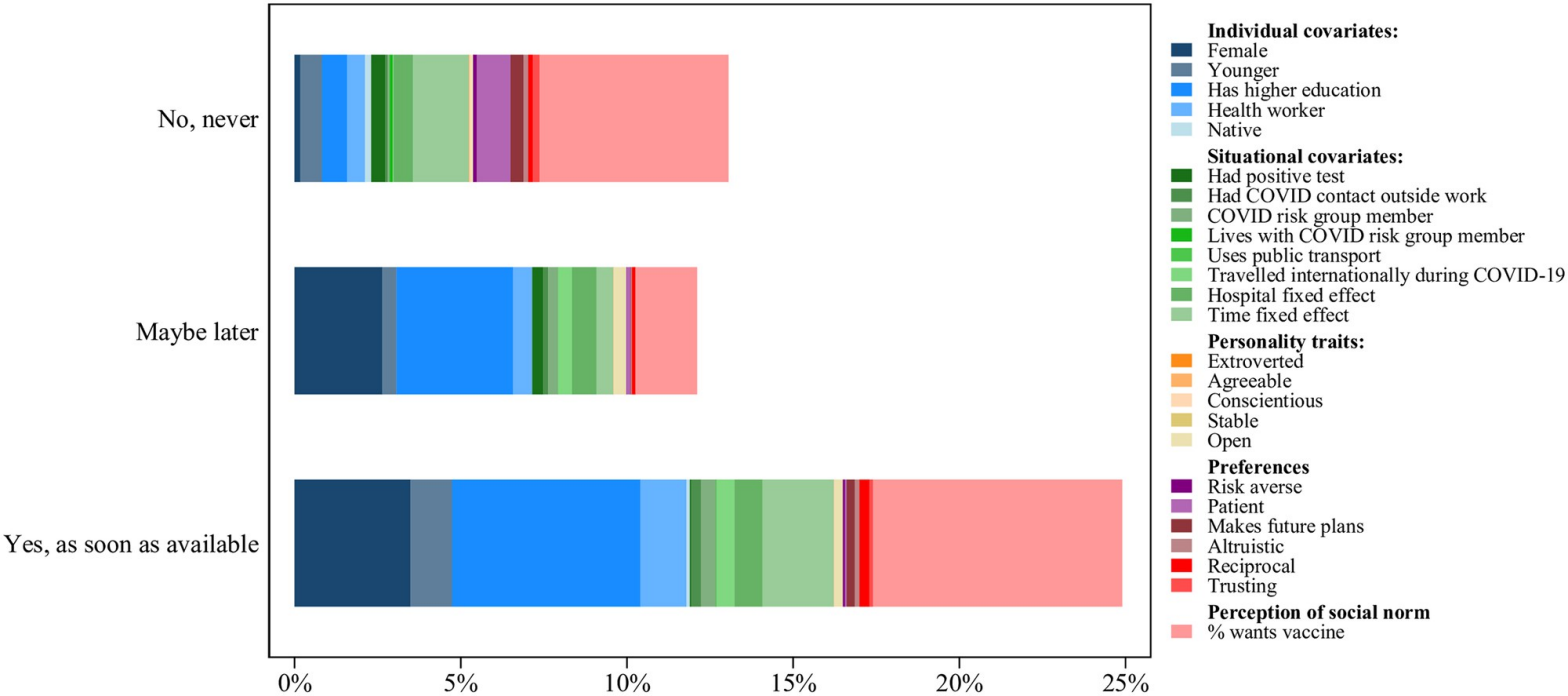
Variance explained by different types of factors. Each row in the figure shows the estimates from a linear probability regression with binary dependent variables. The first dependent variable is equal to 1 if the respondent indicated “No, never”, otherwise 0. The second dependent variable is equal to 1 if the respondent indicated “Maybe later”, otherwise 0. The third dependent variable is equal to 1 if the respondent indicated “Yes, as soon as available”, otherwise 0. All regressions control for the individual characteristics, situational factors, preferences and personality traits. Individual characteristics include being female, being younger, education, being a healthcare worker, and being native. Situational factors include belonging to COVID-19 risk group, living with someone belonging to COVID-19 risk group, having had contact with a COVID-19 infected person outside work, having been outside Switzerland, using public transport and having had a positive test result of COVID-19 in the past. Personality traits include extroversion, agreeableness, conscientiousness, stability and openness. Preferences include the level of trust in strangers, risk aversion, patience, future orientation, altruism and reciprocity and the perceived percentage of people in the region wanting to get vaccinated. All regressions include hospital and time fixed effects.

Fig 8 illustrates how much of the variation in choosing “No, never”, “Maybe later” and “Yes, as soon as available” is explained by different variables. Here, the dependent variable is equal to 1 if the respondent chose the indicated option and 0 otherwise, for each of the three options. Overall, our regression models explain the profile of the respondents who indicate “Yes, as soon as available” better than the profiles of the respondents who indicate “Maybe” or “No”. For the respondents who selected “Yes, as soon as available” or “Maybe”, about two thirds of the variance is explained by individual characteristics, primarily gender and education. For the respondents who selected “No”, these easily observable characteristics explain less than one third of the variation in the dependent variable.

The situational factors seem to be similarly important for all respondents, explaining between one fourth and one third of the variation. Preferences explain about one third of the variation for the respondents who do not want the vaccine, implying that they are more difficult to identify and target from a public policy perspective. Finally, personality traits play a minuscule role.

Similar to the regression results, adding the perceived social norm to the regression explains a major share of the variation in the willingness to get vaccinated—between 40% and 50% for those who prefer not to get vaccinated and 30% for those who want to get vaccinated. Expectedly, it is less predictive for the respondents who are unsure, explaining about 15% of the variation in their willingness to get vaccinated.

## Discussion

Widespread hesitancy about the COVID-19 vaccination appears to be a major obstacle to ending the pandemic. In this study, we set out to understand the behavioral determinants of vaccine hesitancy among hospital employees in Switzerland. Healthcare workers bridge the gap between policymakers and patients and tend to be informal opinion leaders regarding medical matters to their peers. While resistance to vaccinations is not a new phenomenon and the WHO recognized vaccine hesitancy as a top threat to global health even before the COVID-19 pandemic [8, 70–72], vaccine-hesitant healthcare workers can have a substantial influence on the vaccine decisions of the general population. Thus, in the context of the COVID-19 pandemic, healthcare workers can have both direct and indirect influence on the length and extent of the current pandemic situation in many countries around the world.

As policy makers around the world observe the slowing down of the vaccination rates, an important question is to what extent the different factors explain the variation in hospital employees’ decision regarding the COVID-19 vaccine. In line with previous studies, we confirm that gender, age, having higher education and being a healthcare worker are significantly correlated with the willingness to get vaccinated [3, 46–59]. We further show that having been infected by COVID-19, being a COVID-19 risk group member, and having recently traveled abroad are enabling factors associated with higher vaccine willingness. Heterogeneity in vaccine uptake is thus caused by a mix of distinguishable individual experiences, even before considering less directly observable individual characteristics.

Adding to the existing literature, we proceed to examine a comprehensive set of personality traits and behavioral preferences in relation to vaccine willingness among healthcare workers. We find that in particular those hospital employees who are more patient and who perceive vaccination to be the prevailing social norm are more likely to be vaccine acceptant. An open question that remains is whether we see such a strong correlation between the perceived social norm and the willingness to get vaccinated because people who think that many others get vaccinated also want to get vaccinated, or because people who decide to get vaccinated assume that other people in their region behave similarly (social projection).

Meanwhile, Big-5 personality traits seem to play a less robust role in this respect, once controlling for the above-mentioned individual, situational and behavioral factors. As personality traits may be hard to change or observe, this homogeneity between the vaccine-willing and vaccine-hesitant healthcare workers allows for more clearly targeted and tailored messaging. Instead of targeting specific sub-groups in terms of personality traits, future public-health interventions could focus on communicating the true vaccine acceptance rates in the healthcare worker population.

Our findings have several important implications for public-health communication to healthcare workers. One interpretation of our results could imply that targeted communication that only takes into account the sociodemographic correlates of vaccine hesitancy is likely to be insufficient. The messaging needs to be adjusted to take into account the important unobservable preferences and perceived social norms. Furthermore, given that our analysis also sheds light on several stark differences between the hospital employees that are unsure about the COVID-19 vaccine (answering “Maybe”) and against the vaccine (answering “No”), the messaging also needs to be adjusted to the particular subgroup of the vaccine-hesitant hospital workers. Even though the resulting behavior—lack of vaccine uptake—is the same, at least initially, the underlying behavioral drivers appear to differ.

Our results shed some light on the various ongoing interventions around the world that aim to increase vaccine uptake among the general population—from lotteries with varying amounts of cash rewards and goods to nudges and communication campaigns with testimonies from trusted public figures [73–75]. In particular, given the variety of characteristics that play a role in explaining vaccine resistance, it is evident that more diversified approaches within each population are crucial to improve vaccine willingness among hospital employees. To put it frankly, our results hint that there is likely no “one-size-fits-all” solution to the vaccine hesitancy problem.

Our study has several strengths, such as the access to a particularly important sub-population using a particularly wide range of robust and validated measures (including incentivized tools for preference elicitation), but also some notable limitations. Firstly, while our results can lend themselves in identifying and addressing communication gaps among hospital workers, our insights are based on a limited number of respondents (especially in the “No, never” sub-sample) and our results are only correlational in nature, not necessarily implying causation. Secondly, while our results can inform messaging techniques, we are not testing the impact of any particular messaging approach per se. Thirdly, while our study design includes a wide range of potential reasons for vaccine willingness, the list could be expanded even further to include, for example, measures of cultural myths [76] and beliefs about the safety [77] and provenience of the vaccine [78].

## Supporting information

S1 Appendix The supplementary materials provided separately include all survey questions, summary statistics and regression tables for the analyses in the paper. (PDF)
