# Supplementary material for: Which hospital workers do (not) want the jab? Behavioral correlates of COVID-19 vaccine willingness among employees of Swiss hospitals
Source: PLoS One. 2022 May 26;17(5):e0268775. doi: 10.1371/journal.pone.0268775 (PMC9135270; doi:10.1371/journal.pone.0268775)
Supplement: S1 Appendix — (PDF) [file pone.0268775.s001.pdf]

Which hospital workers do (not) want the jab?
Behavioral correlates of COVID-19 vaccine willingness among employees of Swiss hospitals

Ankush Asri[1,2◑], Viola Asri[1,2◑], Baiba Renerte[1,2,3◑], Franziska Föllmi-Heusi[4,5], Joerg D. Leuppi[6,7], Juergen Muser[6], Reto Nüesch[4,7], Dominik Schuler[4,7], Urs Fischbacher[1,2],

1 University of Konstanz, Konstanz, Germany
2 Thurgau Institute of Economics, Kreuzlingen, Switzerland
3 University of Zurich, Zurich, Switzerland
4 Spital Schwyz, Schwyz, Switzerland
5 Swiss Institute for International Economics and Applied Economic Research, University of St.Gallen, Switzerland
6 Kantonsspital Baselland, Liestal, Switzerland
7 University of Basel, Basel, Switzerland

◑These authors contributed equally to this work.

* urs.fischbacher@uni-konstanz.de

Supplementary material

In this document, we present all survey questions, summary statistics and regression tables related to the analysis in the main text.

1 Survey questions

Table S1 below shows the details for all the variables we use in the analyses, the survey questions corresponding to these variables and how they are coded in the analyses. All the preferences and personality traits variables are standardized with respect to mean such that the standard score is $z = \frac{x_i - \mu}{\sigma}$.

Table S1: Survey questions for all variables

**Survey question**	**Scale**	**Coded as**
**Dependent variable**		
Willingness to get vaccinated		
Assuming that the COVID-19 vaccination is available free of charge and approved in Switzerland, would you like to be vaccinated?	Yes, as soon as it is available<br>Maybe later when I have enough information<br>No, never<br>No response	1<br>2<br>3<br>dropped

**Independent variables of interest**		
**Individual covariates**		
Female		
Please indicate your gender:	Female<br>Male<br>No answer	1 if Female<br>0 otherwise
Younger		
Please enter your age in years:	Under 24 years<br>25-34 years<br>35-44 years<br>45-54 years<br>55-64 years<br>65 years or older	1 if<br>age $\leq$ median age<br>0 otherwise
Has higher education		
What is your highest level of education attained?	No education<br>or primary school<br>Realschule<br>(type of secondary school)<br>Secondary school<br>High school<br>Apprenticeship<br>Vocational<br>Matura<br>Higher technical school<br>University of Applied Sciences<br>University	1 if University of Applied Sciences/University<br>0 otherwise
Health care workers		
What is your professional activity in the hospital?	Doctors<br>Nursing staff<br>Administration<br>Technical support<br>Other support	1 if Doctors or Nursing Staff<br>0 otherwise
Swiss native		
Were you born in Switzerland?	Yes<br>No	1 if Yes<br>0 otherwise

Situational covariates		
Had COVID-19 infection		
Have you already had a COVID-19 test performed?	Yes, positive<br>Yes, negative<br>No	1 if Yes, positive<br>0 otherwise
Had COVID-19 contact outside work		
Have you been in close contact outside of work with a person who tested positive for COVID-19?	Yes<br>No<br>Do not know	1 if Yes<br>0 otherwise
COVID-19 risk group member		
Do you count yourself in a COVID-19 risk group?	Yes<br>No<br>Do not know<br>Not specified	1 if Yes<br>0 otherwise
Lives with COVID-19 risk group member		
Do you live in a household with someone who is in a COVID-19 risk group?	Yes<br>No<br>Do not know<br>Not specified	1 if Yes<br>0 otherwise
Uses public transport		
How do you currently go to work?	On foot or bicycle<br>Car or Motorcycle<br>Public transport<br>Work from home	1 if Public transport<br>0 otherwise
Travelled internationally during COVID-19		
Have you travelled abroad for at least 2 days since July 1, 2020?	Yes<br>No	1 if Yes<br>0 otherwise

Personality traits		
Below you will find a number of personality traits that apply to you to a greater or lesser extent. For each statement, please mark the extent to which it applies to you or not. You are to make this classification for pairs of traits in each case, even though one trait may apply more strongly than the other.		
Extrovert		
Extroverted, enthusiastic<br>Restrained, quiet	Likert-scale from 1 to 7<br>Likert-scale from 1 to 7	Average of the first and the reverse of the second variable and standardized
Agreeable		
Understanding, warm-hearted<br>Critical, argumentative	Likert-scale from 1 to 7<br>Likert-scale from 1 to 7	Average of the first and the reverse of the second variable and standardized
Conscientious		
Reliable, self-disciplined<br>Disorganized, careless	Likert-scale from 1 to 7<br>Likert-scale from 1 to 7	Average of the first and the reverse of the second variable and standardized
Stable		
Serene, emotionally stable<br>Anxious, easily upset	Likert-scale from 1 to 7<br>Likert-scale from 1 to 7	Average of the first and the reverse of the second variable and standardized
Open		
Open to new experiences, multi-layered<br>Conventional, uncreative	Likert-scale from 1 to 7<br>Likert-scale from 1 to 7	Average of the first and the reverse of the second variable and standardized

<table>
<tr><th colspan="3">Preferences</th></tr>
<tr><td colspan="3">Risk averse</td></tr>
<tr><td>You are now participating in a dice game where you roll the dice and receive a payout in return. So the dice decides how much money you get. You can choose from 9 games which one you want to play. The games differ in the payout. In the options below, you can see how the 9 games look like:
- On the left is what you get when the dice comes up 1, 2 or 3. - On the right is what you get when the dice comes up 4, 5 or 6.</td><td>80 CHF or 80 CHF
70 CHF or 95 CHF
60 CHF or 110 CHF
50 CHF or 125 CHF
40 CHF or 140 CHF
30 CHF or 155 CHF
20 CHF or 170 CHF
10 CHF or 185 CHF
0 CHF or 200 CHF</td><td>1
2
3
4
5
6
7
8
9
Reversed and standardized</td></tr>
<tr><td colspan="3">Patient</td></tr>
<tr><td>You can choose to receive a payment within a month or a higher payment in 6-7 months. We will now name five situations. Payment within one month is identical in all these situations. The payment in 6-7 months is different in each situation. For each of these situations, we would like to know what you choose.</td><td>200 CHF or 205 CHF
200 CHF or 213 CHF
200 CHF or 226 CHF
200 CHF or 239 CHF
200 CHF or 250 CHF</td><td>5 if always choosing payment later
4 if choosing payment now only in the 1st choice
3 if choosing payment now in the 1st and 2nd choice
2 if choosing payment now in the 1st, 2nd and 3rd choice
1 if choosing payment now in all but last choice
0 if choosing payment now always
Standardized</td></tr>
<tr><td colspan="3">Future oriented</td></tr>
<tr><td>Do you usually make plans for the future or just take each day as it comes?</td><td>Likert-scale from 0 to 10</td><td>Standardized</td></tr>
</table>

	Altruistic	
How well does this statement describe you as a person: "I am willing to donate to good causes without expecting anything in return."	Likert-scale from 0 to 10	Standardized
	Reciprocal	
How well does this statement describe you as a person: " If someone does me a favor, I'm ready to return it."	Likert-scale from 0 to 10	Standardized
	Trusting	
Do you generally assume that most people can be trusted, or do you tend to think that you can't be too careful?	Likert-scale from 0 to 10	Standardized
	Perceives as social norm	
According to your perception, approximately what percentage of people in your region would like to be vaccinated against COVID-19?	0% - 100%	Standardized

2 Summary statistics

Table S2 shows the summary statistics for all variables included in the regression analysis separately for each of the three groups of the respondents choosing "Yes, as soon as it is available", "Maybe later when I have enough information" and "No, never" to indicate their willingness to get vaccinated.

Table S3 shows the summary statistics for all variables included in the regression analysis separately for each of the three groups of the respondents choosing "Yes, as soon as it is available", "Maybe later when I have enough information" and "No, never" to indicate their willingness to get vaccinated.

Table S2: Summary statistics

	mean	sd	min	max
Female	0.783	0.41	0	1
Native Swiss	0.706	0.46	0	1
Age groups:				
Below 24 years	0.089	0.29	0	1
25-34 years	0.266	0.44	0	1
35-44 years	0.233	0.42	0	1
45-54 years	0.201	0.40	0	1
55-64 years	0.202	0.40	0	1
65 years and older	0.008	0.09	0	1
Education levels:				
Low education	0.110	0.31	0	1
Medium education	0.629	0.48	0	1
High education	0.261	0.44	0	1
Occupation:				
Doctors	0.087	0.28	0	1
Nurses	0.513	0.50	0	1
Admininstrative staff	0.148	0.36	0	1
Technical staff	0.048	0.21	0	1
Other staff	0.203	0.40	0	1
Situational covariates:				
Had COVID-19 infection	0.142	0.35	0	1
Had COVID-19 contact outside work	0.204	0.40	0	1
COVID-19 risk group member	0.118	0.32	0	1
Lives with COVID-19 risk group	0.206	0.40	0	1
Uses public transport	0.210	0.41	0	1
Travelled internationally	0.432	0.50	0	1
Preferences:				
Risk averse	4.281	2.84	0	8
Patient	2.496	1.99	0	5
Future oriented	6.244	2.16	0	10
Altruistic	6.372	2.27	0	10
Reciprocal	8.296	1.60	0	10
Trusting	5.791	2.14	0	10
Perceives as social norm	52.530	21.19	0	100
Personality traits:				
Extrovert	4.641	1.24	1	7
Agreeable	5.470	0.92	3	7
Conscientious	5.944	0.94	2	7
Stable	5.225	1.10	2	7
Open	5.262	1.05	2	7
Observations	964			

Table S3: Summary statistics

	Yes				Maybe later				No never				No response			
	mean	sd	min	max	mean	sd	min	max	mean	sd	min	max	mean	sd	min	max
Individual covariates:																
Female	0.684	0.47	0	1	0.850	0.36	0	1	0.831	0.38	0	1	0.898	0.31	0	1
Younger	0.534	0.50	0	1	0.634	0.48	0	1	0.723	0.45	0	1	0.510	0.51	0	1
Has higher education	0.399	0.49	0	1	0.169	0.37	0	1	0.145	0.35	0	1	0.082	0.28	0	1
Healthcare worker	0.642	0.48	0	1	0.580	0.49	0	1	0.518	0.50	0	1	0.510	0.51	0	1
Native Swiss	0.653	0.48	0	1	0.732	0.44	0	1	0.807	0.40	0	1	0.796	0.41	0	1
Situational covariates:																
Had COVID-19 infection	0.130	0.34	0	1	0.159	0.37	0	1	0.060	0.24	0	1	0.143	0.35	0	1
Had COVID-19 contact outside work	0.155	0.36	0	1	0.238	0.43	0	1	0.241	0.43	0	1	0.163	0.37	0	1
COVID-19 risk group member	0.137	0.34	0	1	0.095	0.29	0	1	0.084	0.28	0	1	0.163	0.37	0	1
Lives with COVID-19 risk group	0.187	0.39	0	1	0.211	0.41	0	1	0.169	0.38	0	1	0.347	0.48	0	1
Uses public transport	0.215	0.41	0	1	0.209	0.41	0	1	0.217	0.41	0	1	0.245	0.43	0	1
Travelled internationally	0.508	0.50	0	1	0.385	0.49	0	1	0.386	0.49	0	1	0.327	0.47	0	1
Preferences:																
Risk averse	4.503	2.91	0	8	4.207	2.78	0	8	3.831	2.93	0	8	4.143	2.52	0	8
Patient	2.655	1.96	0	5	2.544	2.01	0	5	1.892	1.98	0	5	2.143	1.85	0	5
Future oriented	6.482	2.11	0	10	6.200	2.09	0	10	5.795	2.49	0	10	5.469	2.03	2	10
Altruistic	6.528	2.24	0	10	6.361	2.21	0	10	6.000	2.53	1	10	5.714	2.39	0	10
Reciprocal	8.484	1.32	3	10	8.207	1.74	0	10	7.976	1.83	2	10	8.327	1.82	4	10
Trusting	5.984	2.08	0	10	5.720	2.15	0	10	5.386	2.35	0	10	5.286	2.22	0	9
Perceives as social norm	61.601	17.66	0	100	48.499	20.54	0	100	33.735	22.05	0	90	46.796	20.49	3	80
Personality traits:																
Extrovert	4.602	1.24	1	7	4.671	1.21	2	7	4.620	1.25	2	7	4.602	1.21	2	7
Agreeable	5.392	0.88	3	7	5.510	0.93	3	7	5.548	0.95	3	7	5.704	0.92	4	7
Conscientious	5.892	0.97	2	7	5.970	0.89	3	7	6.024	0.89	4	7	5.969	1.03	3	7
Stable	5.240	1.12	2	7	5.179	1.08	2	7	5.452	1.08	2	7	4.990	1.15	3	7
Open	5.224	1.07	2	7	5.329	1.00	2	7	5.187	1.02	3	7	5.173	1.18	2	7
Observations	386				421				83				49			

3 Multinomial Probit regressions

Further, we run multinomial probability regressions for the individual characteristics, situational characteristics, standardized preferences and standardized personality traits.

Table S4: Marginal effects from multinomial probit regression for individual covariates

	(1) Yes	(2) Maybe later	(3) No, never
Female	-0.142***	0.138***	0.00397
	(0.0470)	(0.0472)	(0.0140)
Younger	-0.0874**	0.0680*	0.0193*
	(0.0398)	(0.0393)	(0.0117)
Has higher education	0.274***	-0.257***	-0.0172
	(0.0435)	(0.0439)	(0.0139)
Healthcare worker	0.135***	-0.117***	-0.0181*
	(0.0390)	(0.0383)	(0.0101)
Native Swiss	0.0222	-0.0302	0.00808
	(0.0418)	(0.0415)	(0.0130)
Situational covariates	Yes	Yes	Yes
Personality traits	Yes	Yes	Yes
Preferences	Yes	Yes	Yes
Observations	915	915	915

Dependent variable is willingness to get vaccinated. Situational covariates include belonging to COVID-19 risk group, living with someone belonging to COVID-19 risk group, having had contact with a COVID-19 infected person outside work, having been outside Switzerland, using public transport and having had a positive test result of COVID-19 in the past. Preferences include the level of trust in strangers, risk aversion, impatience, propensity to be future oriented, altruism and reciprocity. Personality traits include extroversion, agreeableness, conscientiousness, stability and openness. All regressions include hospital and time fixed effects. Standard errors in parentheses.

* $p < 0.1$, ** $p < 0.05$, *** $p < 0.01$

Table S5: Marginal effects from multinomial probit regression for situational covariates

	(1)<br>Yes	(2)<br>Maybe later	(3)<br>No, never
Had COVID-19 infection	-0.00915	0.0644	-0.0553***
	(0.0544)	(0.0537)	(0.0191)
Had COVID-19 contact outside work	-0.0722	0.0556	0.0167
	(0.0461)	(0.0451)	(0.0133)
COVID-19 risk group member	0.151**	-0.138**	-0.0133
	(0.0610)	(0.0597)	(0.0180)
Lives with COVID-19 risk group member	0.00841	0.0114	-0.0198
	(0.0471)	(0.0460)	(0.0138)
Uses public transport	-0.0422	0.0164	0.0258**
	(0.0460)	(0.0451)	(0.0128)
Travelled internationally during COVID-19	0.0749**	-0.0725**	-0.00239
	(0.0373)	(0.0368)	(0.0111)
Individual covariates	Yes	Yes	Yes
Personality traits	Yes	Yes	Yes
Preferences	Yes	Yes	Yes
Observations	915	915	915

Dependent variable is willingness to get vaccinated. Individual covariates include being female, being younger, education, being a healthcare worker, and being native. Preferences include the level of trust in strangers, risk aversion, impatience, propensity to be future oriented, altruism and reciprocity. Personality traits include extroversion, agreeableness, conscientiousness, stability and openness. All regressions include hospital and time fixed effects. Standard errors in parentheses.

* $p < 0.1$, ** $p < 0.05$, *** $p < 0.01$

Table S6: Marginal effects from multinomial probit regression for personality traits

	(1) Yes	(2) Maybe later	(3) No, never
Extroverted	-0.00665	0.00524	0.00140
	(0.0203)	(0.0198)	(0.0059)
Agreeable	0.00600	-0.00210	-0.00390
	(0.0199)	(0.0197)	(0.0058)
Conscientious	-0.0193	0.0138	0.00549
	(0.0202)	(0.0199)	(0.0060)
Stable	0.0103	-0.0214	0.0111*
	(0.0200)	(0.0196)	(0.0067)
Open	-0.0393*	0.0423**	-0.00298
	(0.0209)	(0.0205)	(0.0058)
Individual covariates	Yes	Yes	Yes
Situational covariates	Yes	Yes	Yes
Preferences	Yes	Yes	Yes
Observations	915	915	915

Dependent variable is willingness to get vaccinated. Individual covariates include being female, being younger, education, being a healthcare worker, and being native. Situational covariates include belonging to COVID-19 risk group, living with someone belonging to COVID-19 risk group, having had contact with a COVID-19 infected person outside work, having been outside Switzerland, using public transport and having had a positive test result of COVID-19 in the past. Preferences include the level of trust in strangers, risk aversion, impatience, propensity to be future oriented, altruism and reciprocity. All regressions include hospital and time fixed effects. Standard errors in parentheses.

* $p < 0.1$, ** $p < 0.05$, *** $p < 0.01$

Table S7: Marginal effects from multinomial probit regression for preferences

	(1)<br>Yes	(2)<br>Maybe later	(3)<br>No, never
Risk averse	0.00531	-0.00508	-0.000234
	(0.0186)	(0.0184)	(0.0051)
Patient	0.00439	0.0121	-0.0165***
	(0.0182)	(0.0179)	(0.0055)
Future oriented	0.0193	-0.0112	-0.00808
	(0.0189)	(0.0185)	(0.0054)
Altruistic	0.0183	-0.0104	-0.00787
	(0.0198)	(0.0195)	(0.0054)
Reciprocal	0.0365*	-0.0306	-0.00585
	(0.0203)	(0.0195)	(0.0052)
Trusting	0.00778	-0.00115	-0.00662
	(0.0201)	(0.0196)	(0.0054)
Perceives as social	0.178***	-0.133***	-0.0446***
norm	(0.0201)	(0.0197)	(0.0075)
Individual covariates	Yes	Yes	Yes
Situational covariates	Yes	Yes	Yes
Personality traits	Yes	Yes	Yes
Observations	915	915	915

Dependent variable is willingness to get vaccinated. Individual covariates include being female, being younger, education, being a healthcare worker, and being native. Situational covariates include belonging to COVID-19 risk group, living with someone belonging to COVID-19 risk group, having had contact with a COVID-19 infected person outside work, having been outside Switzerland, using public transport and having had a positive test result of COVID-19 in the past. Personality traits include extroversion, agreeableness, conscientiousness, stability and openness. All regressions include hospital and time fixed effects. Standard errors in parentheses.

* $p < 0.1$, ** $p < 0.05$, *** $p < 0.01$

4 Robustness checks

4.1 Multinomial Probit regressions with and without controls

First, we show the regressions for the individual characteristics, situational characteristics, standardized preferences and standardized personality traits with and without controls.

Table S8: Marginal effects from multinomial probit regression for individual covariates

	Yes		Maybe later		No, never	
	(1)	(2)	(3)	(4)	(5)	(6)
Female	-0.167***	-0.142***	0.158***	0.138***	0.00977	0.00397
	(0.0433)	(0.0470)	(0.0436)	(0.0472)	(0.0193)	(0.0140)
Younger	-0.138***	-0.0874**	0.0946***	0.0680*	0.0432***	0.0193*
	(0.0365)	(0.0398)	(0.0361)	(0.0393)	(0.0159)	(0.0117)
Has higher education	0.304***	0.274***	-0.253***	-0.257***	-0.0512***	-0.0172
	(0.0409)	(0.0435)	(0.0411)	(0.0439)	(0.0189)	(0.0139)
Healthcare worker	0.140***	0.135***	-0.107***	-0.117***	-0.0327**	-0.0181*
	(0.0370)	(0.0390)	(0.0364)	(0.0383)	(0.0146)	(0.0101)
Native Swiss	-0.0152	0.0222	0.000581	-0.0302	0.0146	0.00808
	(0.0391)	(0.0418)	(0.0389)	(0.0415)	(0.0174)	(0.0130)
Situational covariates	No	Yes	No	Yes	No	Yes
Personality traits	No	Yes	No	Yes	No	Yes
Preferences	No	Yes	No	Yes	No	Yes
Observations	915	915	915	915	915	915

Dependent variable is willingness to get vaccinated. Situational covariates include belonging to COVID-19 risk group, living with someone belonging to COVID-19 risk group, having had contact with a COVID-19 infected person outside work, having been outside Switzerland, using public transport and having had a positive test result of COVID-19 in the past. Preferences include the level of trust in strangers, risk aversion, impatience, propensity to be future oriented, altruism and reciprocity. Personality traits include extroversion, agreeableness, conscientiousness, stability and openness. All regressions include hospital and time fixed effects. Standard errors in parentheses.

* $p < 0.1$, ** $p < 0.05$, *** $p < 0.01$

Table S9: Marginal effects from multinomial probit regression for situational covariates

	Yes		Maybe later		No, never	
	(1)	(2)	(3)	(4)	(5)	(6)
Had COVID-19 infection	0.0574	-0.00915	0.0254	0.0644	-0.0828***	-0.0553***
	(0.0508)	(0.0544)	(0.0504)	(0.0537)	(0.0263)	(0.0191)
Had COVID-19 contact outside work	-0.0912**	-0.0722	0.0740*	0.0556	0.0172	0.0167
	(0.0433)	(0.0461)	(0.0426)	(0.0451)	(0.0183)	(0.0133)
COVID-19 risk group member	0.122**	0.151**	-0.106*	-0.138**	-0.0166	-0.0133
	(0.0537)	(0.0610)	(0.0540)	(0.0597)	(0.0251)	(0.0180)
Lives with COVID-19 risk group member	-0.0205	0.00841	0.0378	0.0114	-0.0173	-0.0198
	(0.0431)	(0.0471)	(0.0425)	(0.0460)	(0.0195)	(0.0138)
Uses public transport	-0.0262	-0.0422	0.0106	0.0164	0.0155	0.0258**
	(0.0422)	(0.0460)	(0.0418)	(0.0451)	(0.0181)	(0.0128)
Travelled internationally during COVID-19	0.117***	0.0749**	-0.102***	-0.0725**	-0.0143	-0.00239
	(0.0340)	(0.0373)	(0.0339)	(0.0368)	(0.0149)	(0.0111)
Individual covariates	No	Yes	No	Yes	No	Yes
Personality traits	No	Yes	No	Yes	No	Yes
Preferences	No	Yes	No	Yes	No	Yes
Observations	915	915	915	915	915	915

Dependent variable is willingness to get vaccinated. Individual covariates include being female, being younger, education, being a healthcare worker, and being native. Preferences include the level of trust in strangers, risk aversion, impatience, propensity to be future oriented, altruism and reciprocity. Personality traits include extroversion, agreeableness, conscientiousness, stability and openness. All regressions include hospital and time fixed effects. Standard errors in parentheses.

* $p < 0.1$, ** $p < 0.05$, *** $p < 0.01$

Table S10: Marginal effects from multinomial probit regression for personality traits

	Yes		Maybe later		No, never	
	(1)	(2)	(3)	(4)	(5)	(6)
Extroverted	-0.00164	-0.00665	0.00331	0.00524	-0.00167	0.00140
	(0.0179)	(0.0203)	(0.0179)	(0.0198)	(0.0078)	(0.0059)
Agreeable	-0.0284	0.00600	0.0241	-0.00210	0.00432	-0.00390
	(0.0178)	(0.0199)	(0.0179)	(0.0197)	(0.0079)	(0.0058)
Conscientious	-0.0194	-0.0193	0.0170	0.0138	0.00238	0.00549
	(0.0180)	(0.0202)	(0.0179)	(0.0199)	(0.0078)	(0.0060)
Stable	0.0254	0.0103	-0.0411**	-0.0214	0.0158*	0.0111*
	(0.0185)	(0.0200)	(0.0184)	(0.0196)	(0.0089)	(0.0067)
Open	-0.0272	-0.0393*	0.0351*	0.0423**	-0.00790	-0.00298
	(0.0187)	(0.0209)	(0.0187)	(0.0205)	(0.0081)	(0.0058)
Individual covariates	No	Yes	No	Yes	No	Yes
Situational covariates	No	Yes	No	Yes	No	Yes
Preferences	No	Yes	No	Yes	No	Yes
Observations	915	915	915	915	915	915

Dependent variable is willingness to get vaccinated. Individual covariates include being female, being younger, education, being a healthcare worker, and being native. Situational covariates include belonging to COVID-19 risk group, living with someone belonging to COVID-19 risk group, having had contact with a COVID-19 infected person outside work, having been outside Switzerland, using public transport and having had a positive test result of COVID-19 in the past. Preferences include the level of trust in strangers, risk aversion, impatience, propensity to be future oriented, altruism and reciprocity. All regressions include hospital and time fixed effects. Standard errors in parentheses.

* $p < 0.1$, ** $p < 0.05$, *** $p < 0.01$

Table S11: Marginal effects from multinomial probit regression for preferences

	Yes		Maybe later		No, never	
	(1)	(2)	(3)	(4)	(5)	(6)
Risk averse	0.0103	0.00531	-0.00939	-0.00508	-0.000938	-0.000234
	(0.0176)	(0.0186)	(0.0173)	(0.0184)	(0.0055)	(0.0051)
Patient	0.0196	0.00439	-0.000923	0.0121	-0.0187***	-0.0165***
	(0.0175)	(0.0182)	(0.0172)	(0.0179)	(0.0063)	(0.0055)
Future oriented	0.0289	0.0193	-0.0201	-0.0112	-0.00880	-0.00808
	(0.0177)	(0.0189)	(0.0173)	(0.0185)	(0.0062)	(0.0054)
Altruistic	0.0130	0.0183	-0.00567	-0.0104	-0.00736	-0.00787
	(0.0184)	(0.0198)	(0.0181)	(0.0195)	(0.0057)	(0.0054)
Reciprocal	0.0330*	0.0365*	-0.0261	-0.0306	-0.00692	-0.00585
	(0.0193)	(0.0203)	(0.0185)	(0.0195)	(0.0062)	(0.0052)
Trusting	0.0250	0.00778	-0.0164	-0.00115	-0.00863	-0.00662
	(0.0186)	(0.0201)	(0.0180)	(0.0196)	(0.0063)	(0.0054)
Perceives as social	0.180***	0.178***	-0.130***	-0.133***	-0.0496***	-0.0446***
norm	(0.0192)	(0.0201)	(0.0187)	(0.0197)	(0.0069)	(0.0075)
Individual covariates	No	Yes	No	Yes	No	Yes
Situational covariates	No	Yes	No	Yes	No	Yes
Personality traits	No	Yes	No	Yes	No	Yes
Observations	915	915	915	915	915	915

Dependent variable is willingness to get vaccinated. Individual covariates include being female, being younger, education, being a healthcare worker, and being native. Situational covariates include belonging to COVID-19 risk group, living with someone belonging to COVID-19 risk group, having had contact with a COVID-19 infected person outside work, having been outside Switzerland, using public transport and having had a positive test result of COVID-19 in the past. Personality traits include extroversion, agreeableness, conscientiousness, stability and openness. All regressions include hospital and time fixed effects. Standard errors in parentheses.

* $p < 0.1$, ** $p < 0.05$, *** $p < 0.01$

4.2 Multinomial Probit regressions with non standardized preferences and personality traits

Second, we show robustness checks where we run multinomial probit regressions for the individual characteristics, situational characteristics, non standardized preferences and non standardized personality traits.

Table S12: Marginal effects from multinomial probit regression for individual covariates

	(1)<br>Yes	(2)<br>Maybe later	(3)<br>No, never
Female	-0.142***	0.138***	0.00397
	(0.0470)	(0.0472)	(0.0140)
Younger	-0.0874**	0.0680*	0.0193*
	(0.0398)	(0.0393)	(0.0117)
Has higher education	0.274***	-0.257***	-0.0172
	(0.0435)	(0.0439)	(0.0139)
Healthcare worker	0.135***	-0.117***	-0.0181*
	(0.0390)	(0.0383)	(0.0101)
Native Swiss	0.0222	-0.0302	0.00808
	(0.0418)	(0.0415)	(0.0130)
Situational covariates	Yes	Yes	Yes
Personality traits	Yes	Yes	Yes
Preferences	Yes	Yes	Yes
Observations	915	915	915

Dependent variable is willingness to get vaccinated. Situational covariates include belonging to COVID-19 risk group, living with someone belonging to COVID-19 risk group, having had contact with a COVID-19 infected person outside work, having been outside Switzerland, using public transport and having had a positive test result of COVID-19 in the past. Preferences include the level of trust in strangers, risk aversion, impatience, propensity to be future oriented, altruism and reciprocity. Personality traits include extroversion, agreeableness, conscientiousness, stability and openness. All regressions include hospital and time fixed effects. Standard errors in parentheses.

* $p < 0.1$, ** $p < 0.05$, *** $p < 0.01$

Table S13: Marginal effects from multinomial probit regression for situational covariates

	(1)<br>Yes	(2)<br>Maybe later	(3)<br>No, never
Had COVID-19 infection	-0.00915	0.0644	-0.0553***
	(0.0544)	(0.0537)	(0.0191)
Had COVID-19 contact outside work	-0.0722	0.0556	0.0167
	(0.0461)	(0.0451)	(0.0133)
COVID-19 risk group member	0.151**	-0.138**	-0.0133
	(0.0610)	(0.0597)	(0.0180)
Lives with COVID-19 risk group member	0.00841	0.0114	-0.0198
	(0.0471)	(0.0460)	(0.0138)
Uses public transport	-0.0422	0.0164	0.0258**
	(0.0460)	(0.0451)	(0.0128)
Travelled internationally during COVID-19	0.0749**	-0.0725**	-0.00239
	(0.0373)	(0.0368)	(0.0111)
Individual covariates	Yes	Yes	Yes
Personality traits	Yes	Yes	Yes
Preferences	Yes	Yes	Yes
Observations	915	915	915

Dependent variable is willingness to get vaccinated. Individual covariates include being female, being younger, education, being a healthcare worker, and being native. Preferences include the level of trust in strangers, risk aversion, impatience, propensity to be future oriented, altruism and reciprocity. Personality traits include extroversion, agreeableness, conscientiousness, stability and openness. All regressions include hospital and time fixed effects. Standard errors in parentheses.
* $p < 0.1$, ** $p < 0.05$, *** $p < 0.01$

Table S14: Marginal effects from multinomial probit regression for personality traits

	(1) Yes	(2) Maybe later	(3) No, never
Extroverted	-0.00537	0.00424	0.00113
	(0.0164)	(0.0160)	(0.0047)
Agreeable	0.00655	-0.00230	-0.00426
	(0.0218)	(0.0215)	(0.0064)
Conscientious	-0.0206	0.0147	0.00584
	(0.0215)	(0.0212)	(0.0063)
Stable	0.00934	-0.0194	0.0100*
	(0.0181)	(0.0178)	(0.0061)
Open	-0.0374*	0.0403**	-0.00284
	(0.0200)	(0.0195)	(0.0055)
Individual covariates	Yes	Yes	Yes
Situational covariates	Yes	Yes	Yes
Preferences	Yes	Yes	Yes
Observations	915	915	915

Dependent variable is willingness to get vaccinated. Individual covariates include being female, being younger, education, being a healthcare worker, and being native. Situational covariates include belonging to COVID-19 risk group, living with someone belonging to COVID-19 risk group, having had contact with a COVID-19 infected person outside work, having been outside Switzerland, using public transport and having had a positive test result of COVID-19 in the past. Preferences include the level of trust in strangers, risk aversion, impatience, propensity to be future oriented, altruism and reciprocity. All regressions include hospital and time fixed effects. Standard errors in parentheses.

* $p < 0.1$, ** $p < 0.05$, *** $p < 0.01$

Table S15: Marginal effects from multinomial probit regression for preferences

	(1) Yes	(2) Maybe later	(3) No, never
Risk averse	0.00187	-0.00179	-0.0000825
	(0.0066)	(0.0065)	(0.0018)
Patient	0.00221	0.00610	-0.00831***
	(0.0092)	(0.0090)	(0.0028)
Future oriented	0.00893	-0.00519	-0.00374
	(0.0088)	(0.0086)	(0.0025)
Altruistic	0.00804	-0.00458	-0.00346
	(0.0087)	(0.0086)	(0.0024)
Reciprocal	0.0228*	-0.0192	-0.00366
	(0.0127)	(0.0122)	(0.0033)
Trusting	0.00364	-0.000540	-0.00310
	(0.0094)	(0.0092)	(0.0025)
Perceives as social norm	0.00840***	-0.00630***	-0.00210***
	(0.0009)	(0.0009)	(0.0004)
Individual covariates	Yes	Yes	Yes
Situational covariates	Yes	Yes	Yes
Personality traits	Yes	Yes	Yes
Observations	915	915	915

Dependent variable is willingness to get vaccinated. Individual covariates include being female, being younger, education, being a healthcare worker, and being native. Situational covariates include belonging to COVID-19 risk group, living with someone belonging to COVID-19 risk group, having had contact with a COVID-19 infected person outside work, having been outside Switzerland, using public transport and having had a positive test result of COVID-19 in the past. Personality traits include extroversion, agreeableness, conscientiousness, stability and openness. All regressions include hospital and time fixed effects. Standard errors in parentheses.

* $p < 0.1$, ** $p < 0.05$, *** $p < 0.01$
